# Genome-wide investigation and expression analysis of *OSCA* gene family in response to abiotic stress in alfalfa

**DOI:** 10.3389/fpls.2023.1285488

**Published:** 2023-11-03

**Authors:** Xiaohong Li, Xiaotong Wang, Xuxia Ma, Wenqi Cai, Yaling Liu, Wenxue Song, Bingzhe Fu, Shuxia Li

**Affiliations:** ^1^ College of Forestry and Prataculture, Ningxia University, Yinchuan, China; ^2^ Inner Mongolia Pratacultural Technology Innovation Center Co., Ltd, Hohhot, China; ^3^ Ningxia Grassland and Animal Husbandry Engineering Technology Research Center, Yinchuan, China; ^4^ Key Laboratory for Model Innovation in Forage Production Efficiency, Ministry of Agriculture and Rural Affairs, Yinchuan, China

**Keywords:** alfalfa, OSCA gene family, genome-wide, expression pattern, abiotic stress

## Abstract

Alfalfa is an excellent leguminous forage crop that is widely cultivated worldwide, but its yield and quality are often affected by drought and soil salinization. Hyperosmolality-gated calcium-permeable channel (OSCA) proteins are hyperosmotic calcium ion (Ca^2+^) receptors that play an essential role in regulating plant growth, development, and abiotic stress responses. However, no systematic analysis of the *OSCA* gene family has been conducted in alfalfa. In this study, a total of 14 *OSCA* genes were identified from the alfalfa genome and classified into three groups based on their sequence composition and phylogenetic relationships. Gene structure, conserved motifs and functional domain prediction showed that all *MsOSCA* genes had the same functional domain DUF221. *Cis*-acting element analysis showed that *MsOSCA* genes had many *cis*-regulatory elements in response to abiotic or biotic stresses and hormones. Tissue expression pattern analysis demonstrated that the *MsOSCA* genes had tissue-specific expression; for example, *MsOSCA12* was only expressed in roots and leaves but not in stem and petiole tissues. Furthermore, RT–qPCR results indicated that the expression of *MsOSCA* genes was induced by abiotic stress (drought and salt) and hormones (JA, SA, and ABA). In particular, the expression levels of *MsOSCA3*, *MsOSCA5*, *MsOSCA12* and *MsOSCA13* were significantly increased under drought and salt stress, and *MsOSCA7*, *MsOSCA10*, *MsOSCA12* and *MsOSCA13* genes exhibited significant upregulation under plant hormone treatments, indicating that these genes play a positive role in drought, salt and hormone responses. Subcellular localization results showed that the MsOSCA3 protein was localized on the plasma membrane. This study provides a basis for understanding the biological information and further functional analysis of the *MsOSCA* gene family and provides candidate genes for stress resistance breeding in alfalfa.

## Introduction

Alfalfa (*Medicago sativa* L.) is a perennial forage grass that is widely grown worldwide and is highly adaptable to the environment. It is known as the ‘king of forages’ due to its high nutritional content and palatability. However, a number of adverse biotic (such as thrips, aphids, brown spot and downy mildew) and abiotic (such as drought, saline-alkali conditions and extreme temperature) factors severely constrain alfalfa production and have a serious negative impact on alfalfa yield and quality (He et al., 2022). For example, drought resulted in poor crop growth due to impeded water and nutrient uptake and excessive salt can lead to crop root death. Therefore, it is necessary to identify superior genes to breed alfalfa varieties with strong environmental adaptability through gene editing.

As an important secondary messenger in plants, calcium ions (Ca^2+^) play a key role in regulating plant growth and development and abiotic stress responses (Kolukisaoglu et al., 2004; Hepler, 2005). Studies have demonstrated that when plants are subjected to osmotic stress, this leads to a temporary increase in Ca^2+^ in the cytoplasm, which triggers the expression of osmotic stress-related genes that regulate plant tolerance to osmotic stress (Zelman et al., 2012; Kaur et al., 2018; Chen et al., 2021). Ca^2+^ mainly relies on calcium channels to enter cells. The major calcium channels in plants include cyclic nucleotide-gated channels (CNGCs) (Galione et al., 2009; Zelman et al., 2012), twin pore channels (TPCS) (Plattner and Verkhratsky, 2015), force-sensitive channels (MCAS), glutamate receptor channels (GLRS) (Hou et al., 2012), and hyperosmolality-gated calcium-permeable channels (OSCA) (Alaimo and Villarroel, 2018). Plants sense calcium signals delivered to effectors via three receptors, calmodulin (CAM) (Batistic and Kudla, 2009; Cheval et al., 2013), calcium-regulated neurophosphatase B-like protein (CBL) (Komatsu et al., 2001; Shabala et al., 2021) and Ca^2+^-dependent protein kinase (CDPK) (Ito et al., 2011; Vivek et al., 2017), and trigger the activation of protective mechanisms. OSCA is a family of calcium-permeable cation channel proteins that respond to hyperosmotic stress (Yuan et al., 2014; Zhang et al., 2023). OSCA family proteins have three conserved functional domains, namely, RSN1_TM (PF13967), DUF4463 (PHM7_cyt, PF14703), and DUF221 (RSN1_7TM, PF02714) (Li et al., 2022). The DUF4463 domain is usually located before the DUF221 domain. The DUF221 structural domain is essential for all *OSCA* family genes, and it is a representative and decisive structural domain that plays a role in the calcium channel during osmosis (Hou et al., 2014).

OSCA is located on the plasma membrane and plays an important role in the regulation of signal transduction during vegetative and reproductive growth in plants (Li et al., 2015). The *OSCA* family genes play specific roles in leaf, flower and root development in *Arabidopsis*, as evidenced by the high expression levels of *Solanum habrochaite OSCA* genes at specific developmental stages (Yuan et al., 2014; Miao et al., 2022). *AtOSCA1.2* is highly permeable to calcium, potassium and sodium ions, which play an important role in ion absorption and exchange (Liu et al., 2018a). In *Arabidopsis*, *OSCA1.3* has the specific function of participating in stomatal closure during immune signal transduction, while *OSCA1.3* does not participate in erythranilic acid-induced stomatal closure (Thor et al., 2020). In wheat, *TaOSCA1.4* is related to the number of grain ears. *TaOSCA1.4* haplotype Hap-1B-C had more spikelets than Hap-1B-A (Lv, 2015). In addition, *OsOSCA1.1/-1.2/-2.4/-3.1/-4.1* play more essential roles in the development of rice roots, buds, mature stems, mature leaves, stamens, pistils and mature seeds, as their expression levels are significantly higher in these tissues than in other tissues (Li et al., 2015).

As the only osmoreceptors in plants, OSCAs are capable of responding to abiotic stress, such as salt and drought, and have been confirmed in a variety of crops, such as rice (Li et al., 2015), sunflower (Shan et al., 2023), cucumber (Yang et al., 2022), soybean (Liu et al., 2022) and maize (Cao et al., 2020). In *Arabidopsis*, *OSCA1* functions as an osmotic sensing receptor that mediates the elevation of Ca^2+^ concentration in response to osmotic stress (Liu et al., 2018b). It was found that silencing the *ShOSCA3* gene decreased the activities of SOD, POD and APX while enhancing the ability of *Solanum habrochaites* to cope with high levels of ABA-induced stress (Miao et al., 2022). Transcriptomic analysis revealed that *OsOSCA1.1* was involved in regulating stomatal closure and promoting seedling survival in rice roots under hyperosmotic and salt stresses (Li et al., 2015). Overexpression of *ZmOSCA2.4* in *Arabidopsis* resulted in upregulation of the *MYB44*, *DREB2A* and *NCED3* genes, while decreasing the expression of senescence-related genes and improving drought tolerance in *Arabidopsis* (Cao et al., 2020). Overall, *OSCA* genes have important roles in plant resistance to high osmotic stress environments. However, little is known about the response of *OSCA* family genes to abiotic stress in alfalfa.

In this study, we identified *OSCA* gene family members in alfalfa by analyzing the evolutionary relationships, gene structure, protein motifs and *cis*-acting elements of *MsOSCA* genes within the whole alfalfa genome. The expression patterns of the *MsOSCA* family members were analyzed in root, stem, leaf and petiole tissues and under salt, drought, abscisic acid (ABA), jasmonic acid (JA) and salicylic acid (SA) stresses. The results of this study lay a foundation for further understanding the functional verification of the *MsOSCA* gene family and provide a reference for exploring the mechanism of *OSCA* genes under abiotic stress in the future.

## Materials and methods

### *OSCA* gene identification in alfalfa

The protein sequences of 15 *Arabidopsis OSCA* genes were downloaded from TAIR. The reference genome of alfalfa (Zhongmu No. 1) and annotation files (https://figshare.com/articles/dataset/Medicago_sativa_genome_and_ annotation_files/12623960) were used in this study. First, 15 AtOSCA protein sequences were used to perform a BLAST search against the alfalfa protein database with the threshold of E-value < 1 × 10 ^−5^ (Chen et al., 2021). Furthermore, the hidden Markov model (HMM) profile of DUF221 was used as a query to search against the alfalfa protein database using the simple HMM search in TBtools (Chen et al., 2020). Subsequently, the results of the BLAST and HMMER searches were merged, and redundancies were removed manually. Then, the Inter Pro (https://www.ebi.ac.uk/interpro/search/sequence/) function was used to confirm whether the candidate MsOSCAs had the conserved DUF221 domain and other typical domains. The gene without the typical functional domain was deleted.

### Basic analysis of MsOSCA proteins

We analyzed the protein length (aa), instability index, molecular weight (MW), isoelectric point (pI) and grand average of hydropathicity (GRAVY) of MsOSCA proteins using the ProtParam ExPASy online website (https://web.expasy.org/protparam/) (Wilkins et al., 1999). SOPMA (https://npsa-prabi.ibcp.fr/cgi-bin/secpred_sopma.pl) was used to analyze the secondary structure of MsOSCA proteins (Ahmad et al., 2021). The subcellular location of MsOSCA proteins was predicted using the online tool WoLF PSORT (https://wolfpsort.hgc.jp) and Cell-PLOC (http://www.csbio.sjtu.edu.cn/bioinf/Cell-PLOC-2/) (Horton et al., 2007). We used the AlphaFold database to analyze the three-dimensional (3D) structure of MsOSCA proteins and PyMOL software to label the typical functional domain (El Khoury et al., 2023).

### Phylogenetic analysis

The phylogenetic tree of 67 OSCA protein sequences, including 14 MsOSCAs from alfalfa, 15 AtOSCAs from *Arabidopsis*, 12 CaOSCAs from *Cicer arietinum*, 13 VrOSCAs from *Vigna radiata* and 13 CcOSCAs from *Cajanus cajan*, was constructed using MEGA 11 software with the maximum likelihood (ML) estimate, 1000 bootstrap replicates and other parameters set to default (Gregory et al., 2017; Yan et al., 2021). 67 OSCA protein sequences on **Supplementary Table S1**.

### Functional domain, conserved motif and gene structure analysis

InterPro (https://www.ebi.ac.uk/interpro/) online website was used to analyze the functional domains of MsOSCA proteins. We used MEME (https://meme-suite.org/meme/tools/meme) to analyze conserved motifs and set the maximum number of predicted patterns to 10, and the screening threshold was E < e^-10^ (Bailey et al., 2009). The gene structure was analyzed using the alfalfa genome annotation files and visualized with TBtools.

### Analysis of promoter *Cis*-Acting element and MsOSCA protein interactions

The 2 kb genomic DNA sequence upstream of the start codon (ATG) of each *MsOSCA* gene was extracted from the alfalfa genome annotation files using TBtools. The *cis*-regulatory elements in the promoter sequences of *MsOSCA* genes were analyzed using PlantCare (http://bioinformatics.psb.ugent.be/webtools/plantcare/html/) (Rombauts et al., 1999; Lescot et al., 2002). *Arabidopsis* was used as a model plant for query, and STRING online website (http://cn.string-db.org/) was used to analyze the protein–protein interactions of MsOSCAs.

### Chromosome location, gene duplication and collinearity analysis

The genomic data for *Arabidopsis* and maize were downloaded from the EnsemblPlants database (https://plants.ensembl.org/index.html). The chromosome distribution of *MsOSCA* genes was analyzed and localized using TBtools software (https://github.com/CJ-Chen/TBtools) (Yin et al., 2021). A multiple collinear scanning toolkit (MCScanX) was utilized to analyze the collinear blocks of *OSCA* genes across alfalfa, *Arabidopsis* and *Medicago truncatula* and visualized by TBtools. Synonymous (Ka), nonsynonymous (Ks) substitutions, and Ka/Ks ratios were calculated by the Simple Ka/Ks Calculator (NG) (Li et al., 2023).

### Analysis of *MsOSCA* gene expression using transcriptome data

The transcriptome data of alfalfa (Xinjiangdaye) were downloaded from the NCBI website (https://www.ncbi.nlm.nih.gov/sra/?term=SRP055547) (O'Rourke et al., 2015). The most closely matching transcript sequences of *MsOSCAs* were identified in the Alfalfa Breeder’s Toolbox database using the BLAST alignment tool (https://www.alfalfatoolbox.org/), and the expression profiles of *MsOSCA* gene*s* in different tissues of alfalfa were obtained. The data for each *MsOSCA* were normalized by logarithm (FPKM) and displayed as a heatmap using TBtools.

### Plant materials and stress treatments

Alfalfa (Zhongmu No. 1) seeds were sterilized with 75% ethanol for 30 s and 50% sodium hypochlorite for 5-6 min, rinsed 10 times with distilled water and incubated on wet filter paper in petri dishes at 25°C. After 3 days of growth in petri dishes, alfalfa seedlings were transferred to a plastic container (25 cm × 18 cm × 14 cm) with Hoagland nutrient solution for hydroponics under controlled conditions: temperature 24 ± 1°C, 16 hours of light, 8 hours of darkness, and 60% relative humidity. After 30 days of growth, the seedlings were exposed to nutrient solutions supplemented with 20% PEG-6000, 200 mM NaCl, 100 μM JA, 100 μM SA, or 100 μM ABA for different treatments (Luo et al., 2019). The seedlings cultured with normal nutrient solution without adding any substance served as the control. Alfalfa leaf samples from the control and treatment groups were collected at five time points of 0, 2, 4, 8, and 12 h for gene expression analysis. The roots, stems, leaves and petioles of the untreated seedlings were collected for gene expression analysis of different tissues. All samples were rapidly frozen in liquid nitrogen and stored at -80°C for further RNA extraction.

### RNA extraction and quantitative real-time PCR analysis

Total RNA was extracted from the samples using the RNA Extraction Kit (Promega, Shanghai, China) according to the instructions. A total of 1 μg RNA was used for reverse transcription, and RT–qPCR was carried out according to the method provided by ChamQ Universal SYBR qPCR Master Mix (Vazyme, Nanjing, China). The *MsActin* gene (Li et al., 2019) was used as an internal reference gene, and all primers used in this study are listed in **Supplementary Table S6**. All primer concentrations were 10 μg/ml. The RT–qPCR system was 20 μl, containing 2 μl of cDNA, 0.6 μl of forward and reverse primers, 10 μl of ChamQ Universal SYBR enzyme and 6.8 μl of ddH_2_O (No RNA enzyme water). The reaction procedure was as follows: 3 min at 95°C; 10 s at 95°C, 30 s at 60°C for 40 cycles; 5 s at 65°C and 5 s at 95°C. Each treatment for RT–qPCR consisted of three independent biological replicates and three technical replicates. The melting curves of all primers used in this study are shown in **Figure S1**. The expression values of root tissue or 0 h were normalized, and the relative expression values of other tissues or time points were evaluated. The relative expression levels of genes were calculated by 2^-ΔΔCT^ (Livak and Schmittgen, 2001).

### Subcellular localization of MsOSCA proteins

Based on the results of *MsOSCA* gene response to abiotic stress, *MsOSCA3* was selected for amplification, and the primers are shown in **Supplementary Table S6**. After PCR product recovery and purification, the CDS clones were inserted into the *Eco*RI cloning site in the pCAMBIA-1300-GFP vector. The recombinant vector was separately transformed into *Agrobacterium tumefaciens* GV3101. To validate the transient expression of *MsOSCAs* in tobacco, *Agrobacterium* carrying the recombinant vector was injected into tobacco leaves grown for one month. After incubation in the dark for 48 h, the leaf epidermal cells were used for microscopic (Leica SP8, Germany) observation and image acquisition by the laser confocal method.

### Statistical analysis

Origin 2023 software was used to test the differences between groups by one-way analysis of variance (ANOVA), and Tukey’s multiple range test was used to assess the significant differences, where *P* < 0.05 indicated significance. Data are expressed as the mean or mean ± standard deviation (SD).

## Results

### Genome-wide identification of OSCA members in alfalfa

To identify all *OSCA* family members in alfalfa, the protein sequences of 15 *OSCA* genes in *Arabidopsis* were downloaded from TAIR as queries to search the alfalfa genome (https://figshare.com/articles/dataset/Medicago_sativa_genome_and_annotation_files/12623960). After removing identical and incomplete gene sequences, a total of 14 *OSCA* genes containing RSN1_7TM (PF02714) conserved functional domains were identified. These members were named *MsOSCA1* to *MsOSCA14* according to their position on the chromosome (**Table 1**). The nucleic acid and protein sequences of *MsOSCA* gene family members are shown in **Supplementary Table S1**. The lengths of MsOSCA protein sequences ranged from 205 (MsOSCA8) to 1265 aa (MsOSCA7), with corresponding protein molecular weights (MWs) of 23.55 to 143.01 kDa. The theoretical isoelectric point (pI) and instability index of MsOSCAs ranged from 5.43~9.32 and 37.03~53.18, respectively, with a GRAVY of (-0.229) ~0.297. Secondary structure analysis of the MsOSCA proteins showed that the α-helix, extended strand, β-turn and random coils ranged from 28.57% (MsOSCA12) to 60.57% (MsOSCA2), 11.79% (MsOSCA2) to 20.40% (MsOSCA6), 1.68% (MsOSCA3) to 9.83% (MsOSCA6), and 26.16% (MsOSCA11) to 46.18% (MsOSCA12), respectively. Subcellular localization prediction of WoLF PSORT showed that most MsOSCA proteins were located on the plasma membrane, whereas MsOSCA5, MsOSCA8, and MsOSCA14 were located in the chloroplast. Subcellular localization predicted using Cell-PLOC showed that only six MsOSCA proteins were located in the cell membrane.

**Table 1:** Information on MsOSCA proteins in alfalfa.

Gene name	Gene ID	Chr	Protein length (aa)	Instability index	MW (kDa)		pI	GRAVY	α- Helix (%)	Extended strand (%)	Beta turn (%)	Random coil (%)	Subcellular localization	
													WoLF PSORT	Cell-PLoc
*MsOSCA1*	MsG0180000365.01.T01	1	549	52.75	61.77		8.85	0.13	51.37	13.30	3.64	31.69	Plas	Chlo
*MsOSCA2*	MsG0180002250.01.T01	1	492	41.05	55.96		8.87	0.07	60.57	11.79	2.24	25.41	Plas	Extr, Chlo
*MsOSCA3*	MsG0280006998.01.T01	2	715	37.52	80.76		9.32	0.30	56.50	12.17	1.68	29.65	Plas	Plas
*MsOSCA4*	MsG0380012240.01.T01	3	605	38.92	68.86		8.62	0.12	53.06	12.89	2.64	31.40	Plas	Chlo, Nucl
*MsOSCA5*	MsG0480022114.01.T01	4	228	40.35	26.06		6.96	0.37	42.98	19.74	2.63	34.65	Chlo	Plas
*MsOSCA6*	MsG0580025389.01.T01	5	804	37.03	90.84		9.08	0.11	33.08	20.40	9.83	36.69	Plas	Pero
*MsOSCA7*	MsG0580025468.01.T01	5	1265	37.72	143.01		8.94	0.16	41.66	19.29	7.98	31.07	Plas	Pero
*MsOSCA8*	MsG0580029452.01.T02	5	205	42.46	23.55		5.76	-0.20	41.46	13.66	2.93	41.95	Chlo	Plas
*MsOSCA9*	MsG0580029468.01.T01	5	1200	35.82	136.20		6.61	0.04	0.42	17.75	5.50	34.67	Plas	Plas, Nucl
*MsOSCA10*	MsG0680030842.01.T01	6	665	45.69	76.30		8.34	0.18	49.32	14.29	2.71	33.68	Plas	Plas, Chlo
*MsOSCA11*	MsG0680034489.01.T01	6	279	53.18	32.43		8.74	0.20	55.20	16.85	1.79	26.16	Plas	Extr
*MsOSCA12*	MsG0780036349.01.T01	7	301	46.99	33.67		5.43	0.04	28.57	17.94	7.31	46.18	Plas	Plas, Chlo
*MsOSCA13*	MsG0880045603.01.T01	8	646	42.20	71.82		8.72	0.11	48.30	13.93	3.56	34.21	Plas	Chlo
*MsOSCA14*	MsG0880047674.01.T01	8	273	45.38	31.15		6.06	-0.23	38.83	19.05	6.96	35.16	Chlo	Nucl

aa, number of amino acids sequence; MW, theoretical molecular weight of proteins; pI, theoretical isoelectric point of proteins; GRAVY, grand average of hydropathicity; Plas, plasma membrane; Chlo, chloroplast; Nucl, Nucleus; Extr, Cell wall; Pero, Peroxisome.

### Phylogenetic analysis of MsOSCA proteins in alfalfa

**Figure 1:**
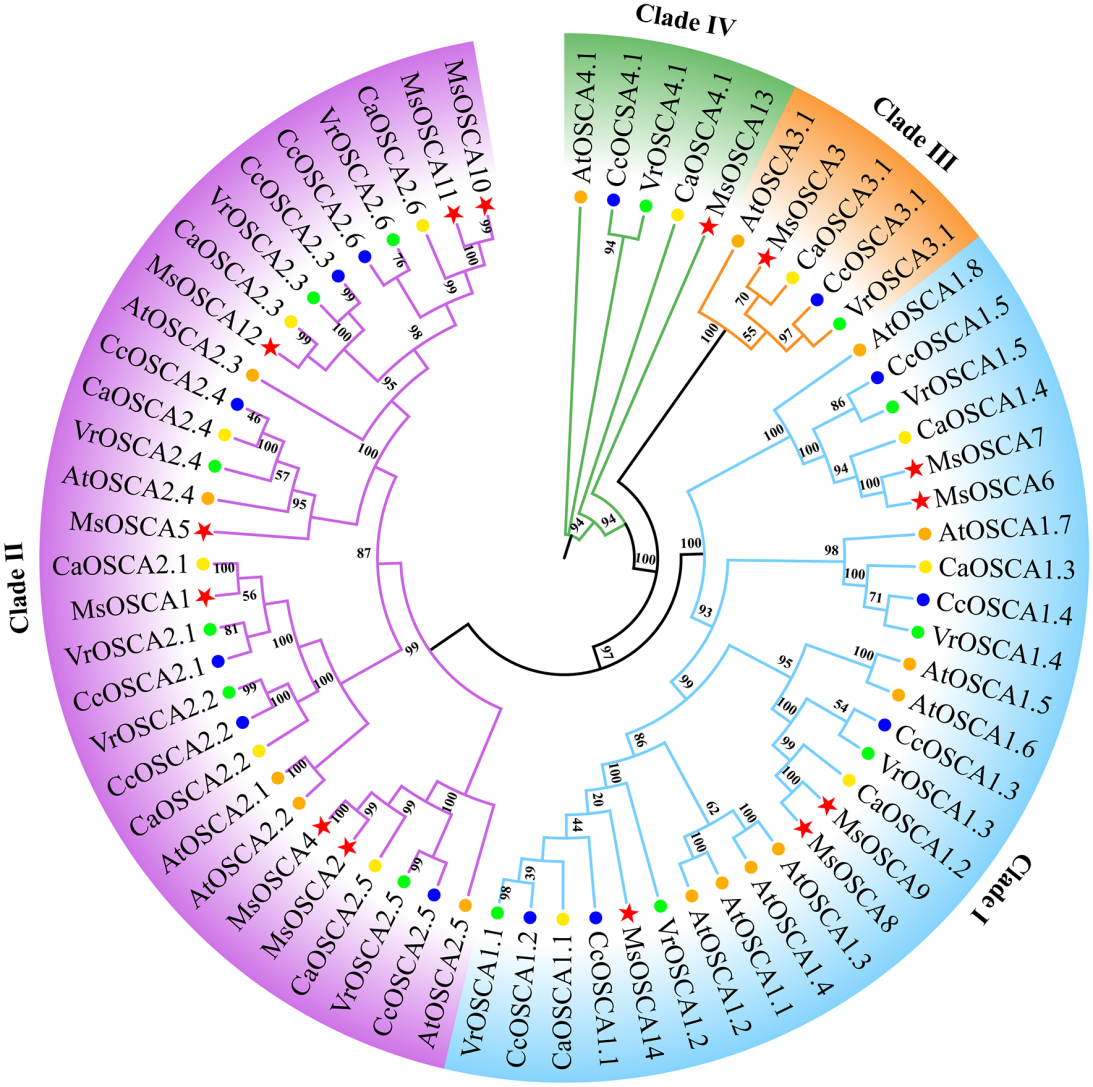
Phylogenetic tree analysis of OSCA proteins in *M. sativa, Arabidopsis, Cicer arietinum, Vigna radiata and Cajanus cajan*. The phylogenetic tree was constructed using OSCA protein sequences by the maximum likelihood estimate (ML) in MEGA 11 with 1000 bootstrap replicates. The red stars, blue circles, green circles, yellow circles and orange circles represent the *M. sativa*, *Cajanus cajan*, *Vigna radiata*, *Cicer arietinum*, and *A. thaliana* OSCA proteins, respectively.

To investigate the evolutionary relationships of OSCA proteins, a phylogenetic tree was constructed using OSCA family members from *M. sativa*, *Arabidopsis*, *Cicer arietinum*, *Vigna radiata* and *Cajanus cajan*. As shown in **Figure 1**, a total of 67 OSCA proteins were classified into four clades (I, II, III and IV) based on the similarity of the full-length sequences. Clade I consisted of 8 AtOSCAs, 5 MsOSCAs, 5 CcOSCAs, 5 VrOSCAs and 4 CaOSCAs. Clade II consisted of 5 AtOSCAs, 7 MsOSCAs, 6 CcOSCAs, 6 VrOSCAs and 6 CaOSCAs. Clade III had 5 OSCA proteins, with one (MsOSCA3) alfalfa members. Clade IV contained one MsOSCA protein (MsOSCA13) of alfalfa. Furthermore, the OSCAs of alfalfa and *Arabidopsis*, *C. arietinum*, *V. radiata* and *C. cajan* were closer together in clades, indicating that they had higher homology. Overall, these results indicated that OSCA family proteins were highly conserved among plant species.

### Functional domains, conserved motifs and gene structure analysis of *MsOSCA* genes

**Figure 2:**
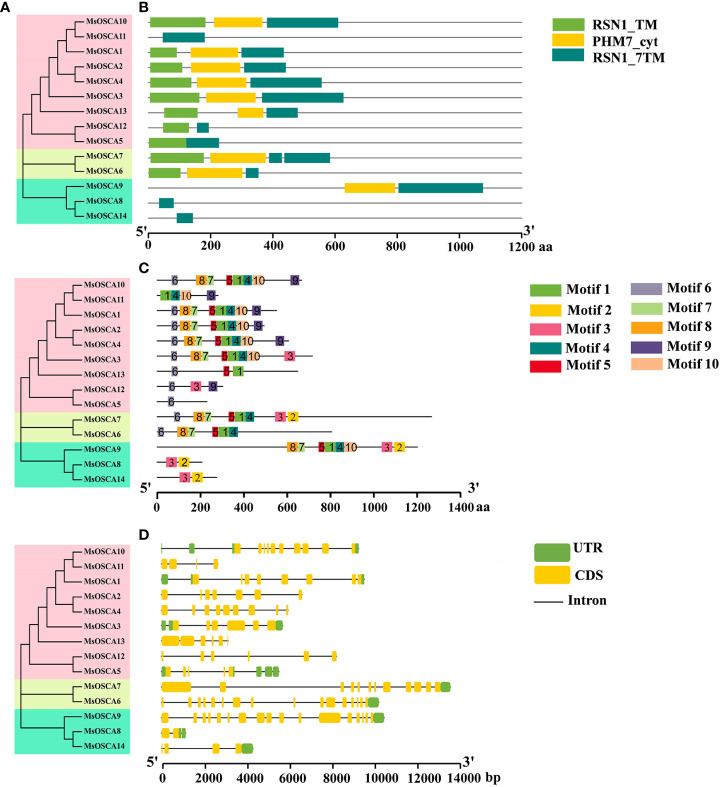
Analysis of the conserved domains, motifs and gene structure of alfalfa *MsOSCA* genes. **(A)** Phylogenetic tree of *MsOSCA* gene family. **(B)** Functional domains distribution of MsOSCAs. The colored rectangles represent the protein conserved domains. **(C)** Motifs of MsOSCA proteins. Different motifs are annotated by boxes of different colors and numbered 1–10. **(D)** Exon-intron structure of *MsOSCA* genes. The untranslated regions (UTRs) are indicated by green boxes. Yellow boxes represent exons and grey lines represent introns.

The RSN1_7TM (PF02714) functional domain is the signature domain of the OSCA family and represents the seven transmembrane functional domains of the calcium-dependent channel (Liu et al., 2018b). All 14 *MsOSCA* members of alfalfa contained the conserved RSN1_7TM (PF02714) functional domain (**Figure 2B**). In addition, MsOSCA1/-2/-3/-4/-6/-7/-9/-10/-13 had RSN1_TM and PHM7_cyt functional domains, and the PHM7_cyt functional domain was located in the middle of the RSN1_TM and RSN1_7TM functional domains. Furthermore, similar conserved structural domains were found in MsOSCA proteins that were closely related on the evolutionary branches (**Figures 2A, B**). The 3D structures of the MsOSCA proteins are shown in **Figure S2**. All MsOSCA proteins contained the PHM7_cyt functional domain, which existed in linear and helical forms. MsOSCA6 and MsOSCA7 were closely related in the evolutionary tree, and both contained the AMP-binding domain. Interestingly, only MsOSCA14 contained the AUX_IAA structural domain.

To better understand the conserved structure of MsOSCA proteins, the distribution of conserved motifs was identified and visualized using the MEME program and TBtools software, respectively. As shown in **Figure 2C**, a total of 10 conserved motifs were identified in alfalfa MsOSCA proteins. The sequence information for each motif is provided in **Supplementary Table S2** and **Figure S3**. The results showed that the number of motifs contained in the 14 MsOSCAs varied widely. For example, MsOSCA1/-2/-3/-4/-6/-7/-9/-10 contained more than 5 motifs, of which MsOSCA7 contained 9 motifs. In contrast, MsOSCA8 and MsOSCA14 had only two motifs, and MsOSCA5 only contained motif 6. Moreover, MsOSCA1/-2/-4/proteins had the same quantity and type motifs, and correspondingly, they were close to each other on the phylogenetic tree, indicating high homology (**Figure 2A**).

Gene structure plays an important role in the regulation of gene expression (Oudelaar and Higgs, 2020). By analyzing the *MsOSCA* gene structure, we found that all members of the *MsOSCA* gene family had introns and exons, but the number varied (**Figure 2D**). For instance, *MsOSCA9* contained 16 introns and 17 exons, while *MsOSCA8* only contained 1 intron and 2 exons. *MsOSCA12* and *MsOSCA13* contained the same number of introns and exons. In addition, some of the *MsOSCAs* contained UTRs, the number of which varied between 1 and 5. Among them, *MsOSCA6*, *MsOSCA7*, *MsOSCA9* and *MsOSCA14* contained only one UTR, whereas *MsOSCA5* contained five UTRs.

### Analysis of *cis*-acting elements in the *MsOSCA* gene promoters

**Figure 3:**
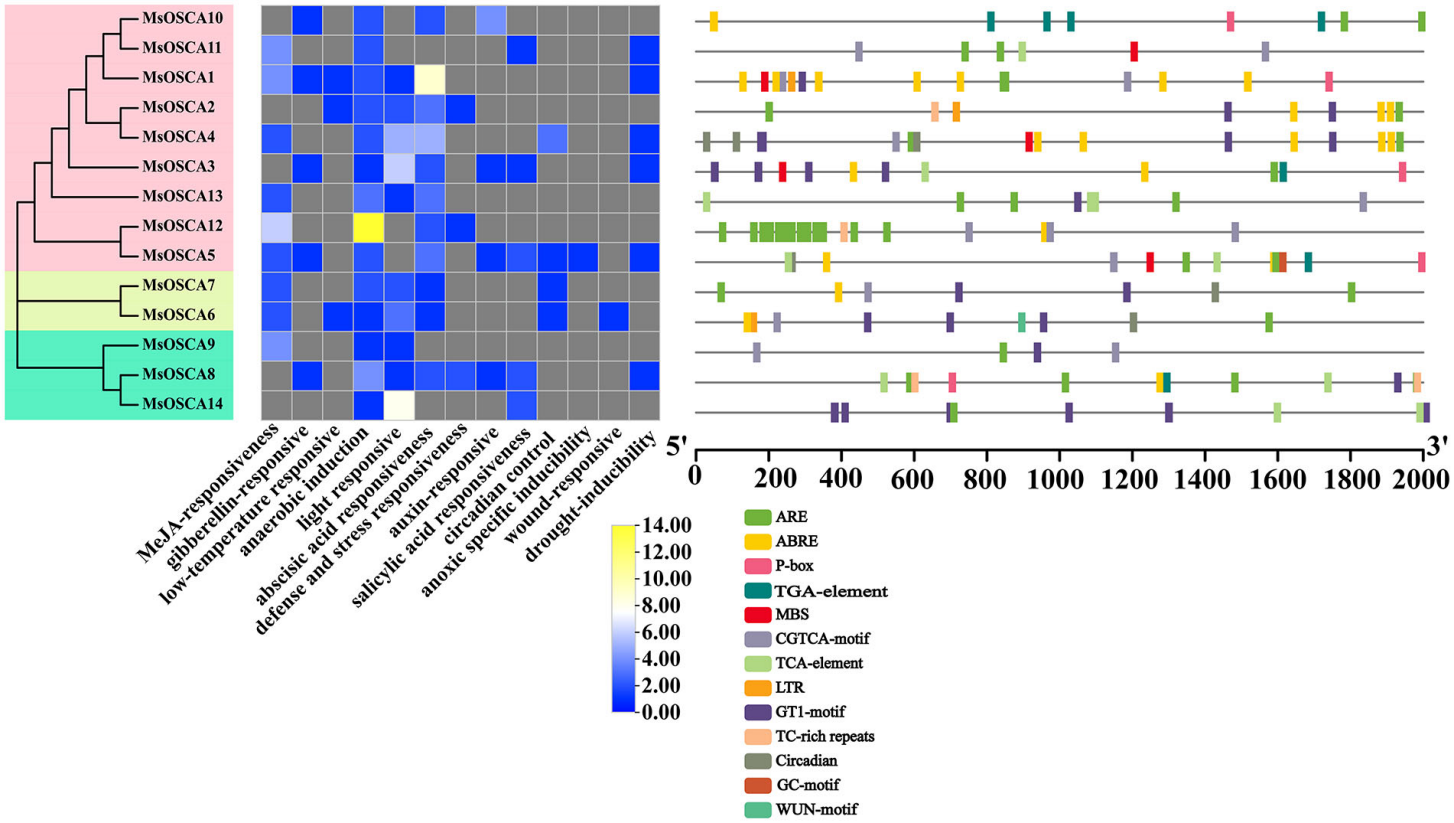
Distribution of *cis*-acting elements in the promoter region of *MsOSCA* genes in alfalfa. The *cis*-elements are represented by rectangles of different colors. ARE: responsive element for the anaerobic induction; ABRE: abscisic acid responsive element; P-box: gibberellin-responsive element; TGA-element: auxin-responsive element; MBS: MYB binding site involved in drought-inducibility; CGTCA-motif: *cis*-acting regulatory element involved in the MeJA-responsiveness; TCA-element: *cis*-acting element involved in salicylic acid responsiveness; LTR: *cis*-acting element involved in low-temperature responsiveness; GT1-motif:light responsive element; TC-rich repeats: *cis*-acting element involved in defense and stress responsiveness; Circadian: *cis*-acting regulatory element involved in circadian control; GC-motif: enhancer-like element involved in anoxic specific inducibility; WUN-motif: wound-responsive element.

The *cis*-acting elements in the promoters of *MsOSCA* members were analyzed by PlantCare. As shown in **Figure 3**, the results showed that a total of 13 different types of *cis*-acting elements were predicted in the *MsOSCA* genes, which were widely involved in growth processes, hormone responsiveness, light responsiveness, metabolic regulation, and stress responses (**Supplementary Table S3**). The number of CGTCA motif, ARE, GT1 motif, TCA element and ABREs was relatively high in the *MsOSCA* genes, followed by GARE motif, TGA element, ABRE, circadian and MBS elements. Of these, stress response-related elements were found in almost all *MsOSCAs*. All 14 *MsOSCAs* contained AREs of stress-related *cis*-elements. Furthermore, the drought response element MBS was found in *MsOSCA1/-3/-4/-5/-11* (**Figure 3**). These results indicated that *MsOSCA* genes may play an active role in the regulation of various stresses and plant hormone responses.

### Analysis of the chromosomal localization and collinearity of *MsOSCA* genes

**Figure 4:**
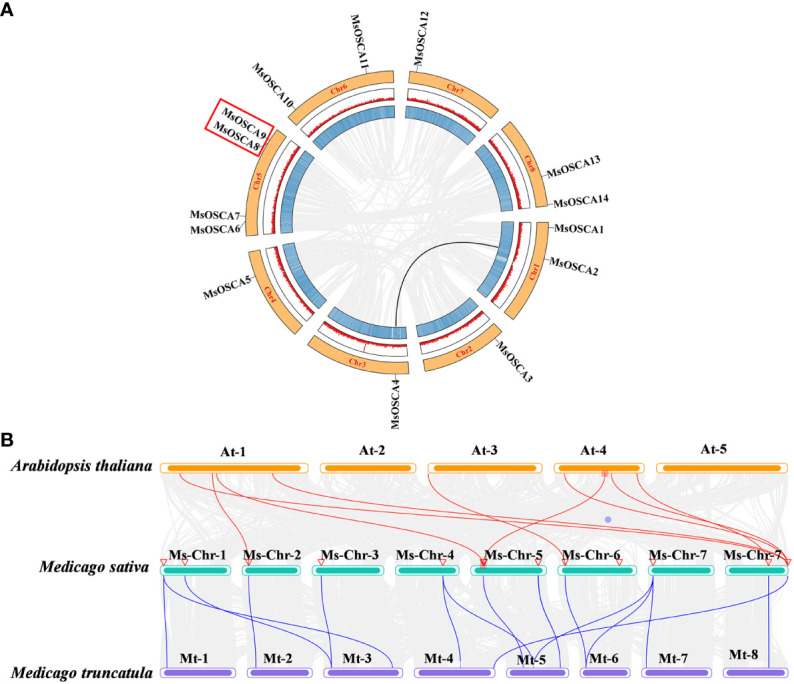
Chromosomal distribution and colinearity analysis of *MsOSCA* genes. **(A)** Chromosomal localization and interchromosomal relationships in alfalfa. The black lines represented the *MsOSCA* collinearity genes. **(B)** Colinearity analysis of *OSCA* genes in alfalfa, *Medicago truncatula* and *Arabidopsis*. The grey lines in the background represent collinear blocks between alfalfa and *Arabidopsis*/*Medicago truncatula*, and the red/blue lines represent collinear *OSCA* gene pairs.

The position of *MsOSCA* genes on each chromosome was determined by alfalfa genome annotation. As shown in **Figure 4A**, 14 *MsOSCA* genes were unevenly distributed on 8 chromosomes of alfalfa. Chromosomes 2, 3, 4 and 7 each contained one *MsOSCA* gene, chromosomes 1, 6 and 8 each contained two *MsOSCA* genes, and chromosome 5 contained four *MsOSCA* genes. To further investigate the evolutionary mechanisms and orthologous relationship of the *MsOSCA* gene family, we analyzed tandem and segmental duplication events. We found that *MsOSCA2* and *MsOSCA4* were segmental duplications, and *MsOSCA8* and *MsOSCA9* were tandem duplications (**Figure 4A**; **Table 2**). In addition, we analyzed the collinearity of *OSCA* genes from alfalfa, *Medicago truncatula* and *Arabidopsis* (**Figure 4B**). Notably, nine direct homologous pairs were found between alfalfa and *Arabidopsis*, and fifteen direct homologous pairs were observed between alfalfa and *Medicago truncatula*. The results showed that eleven *MsOSCA* genes (*MsOSCA1/-2/-3/-4/-5/-6/-8/-9/-10/-12/-14*) had a collinear relationship with *Medicago truncatula*, while five genes (*MsOSCA3/-6/-10/-13/-14*) were found to have a collinear relationship with *Arabidopsis* (**Figure 4B**).

**Table 2:** The divergence between MsOSCA gene pairs in alfalfa.

Paralogous pairs	Ka	Ks	Ka/Ks	Duplicate date (Mya)	Duplicate type
*MsOSCA2/MsOSCA4*	0.067	0.197	0.342	16.171	Segmental
*MsOSCA8/MsOSCA9*	0.130	0.213	0.612	17.469	Tandem

For each gene pair, the Ks value was translated into divergence time in millions of years based on a rate of 6.1×10^−9^ substitutions per site per year. The divergence time (T) was calculated as T= Ks/ (2×6.1×10^−9^) ×10^−6^ Mya.

### Expression patterns of *MsOSCA* genes in different tissues

**Figure 5:**
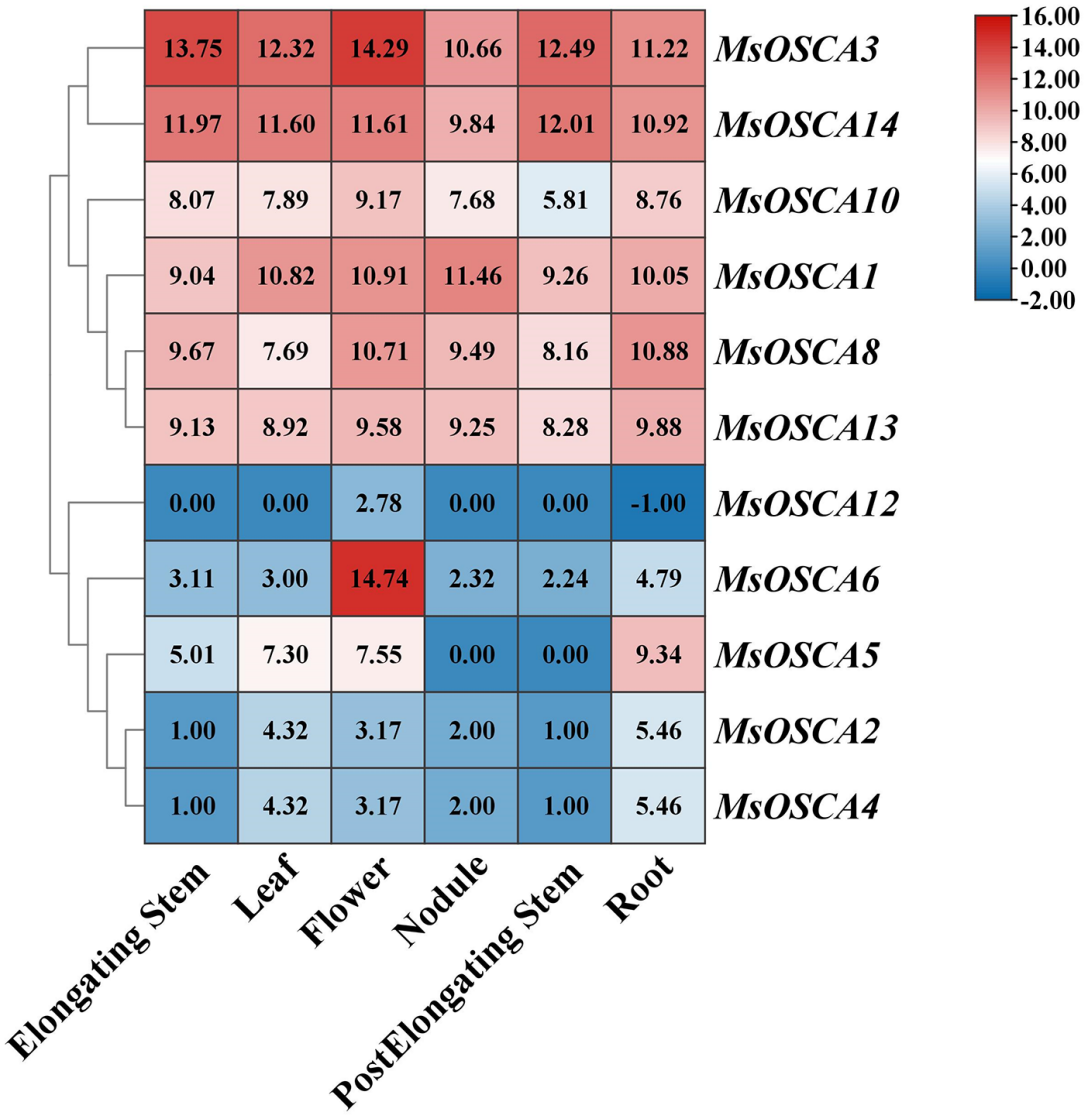
Expression patterns of *MsOSCA* genes in different tissues at various growth stages of alfalfa. The values in the heat map represent the amount of gene expression (FPKM).

To further study the function of *MsOSCAs* in growth and development, we analyzed the expression of *MsOSCA* genes in six different tissues (roots, leaves, flowers, elongating stems, postelongating stems, and nodules) of alfalfa “Xinjiangdaye” based on transcriptomic data (**Supplementary Table S4**). In this study, the expression values of 11 *MsOSCAs* were determined and comprehensively investigated (**Figure 5**). The results showed that the expression of 11 *MsOSCA* genes changed remarkably, and they were obviously divided into two groups. As a whole, *MsOSCA1*, *MsOSCA3*, *MsOSCA8*, *MsOSCA10*, *MsOSCA13* and *MsOSCA14* were highly expressed in all tissues, while other genes had lower expression levels, except for the high expression of *MsOSCA5* in roots and *MsOSCA6* in flowers. The expression levels of *MsOSCA2* and *MsOSCA4* in leaves and roots were significantly higher than those in other tissues. The expression of *MsOSCA12* was high in flowers, indicating that it is a specific expression gene for flower development. *MsOSCA5* was not expressed in nodules or postelongating stems (**Figure 5**). These results suggested that *MsOSCA* family genes had tissue-specific expression.

**Figure 6:**
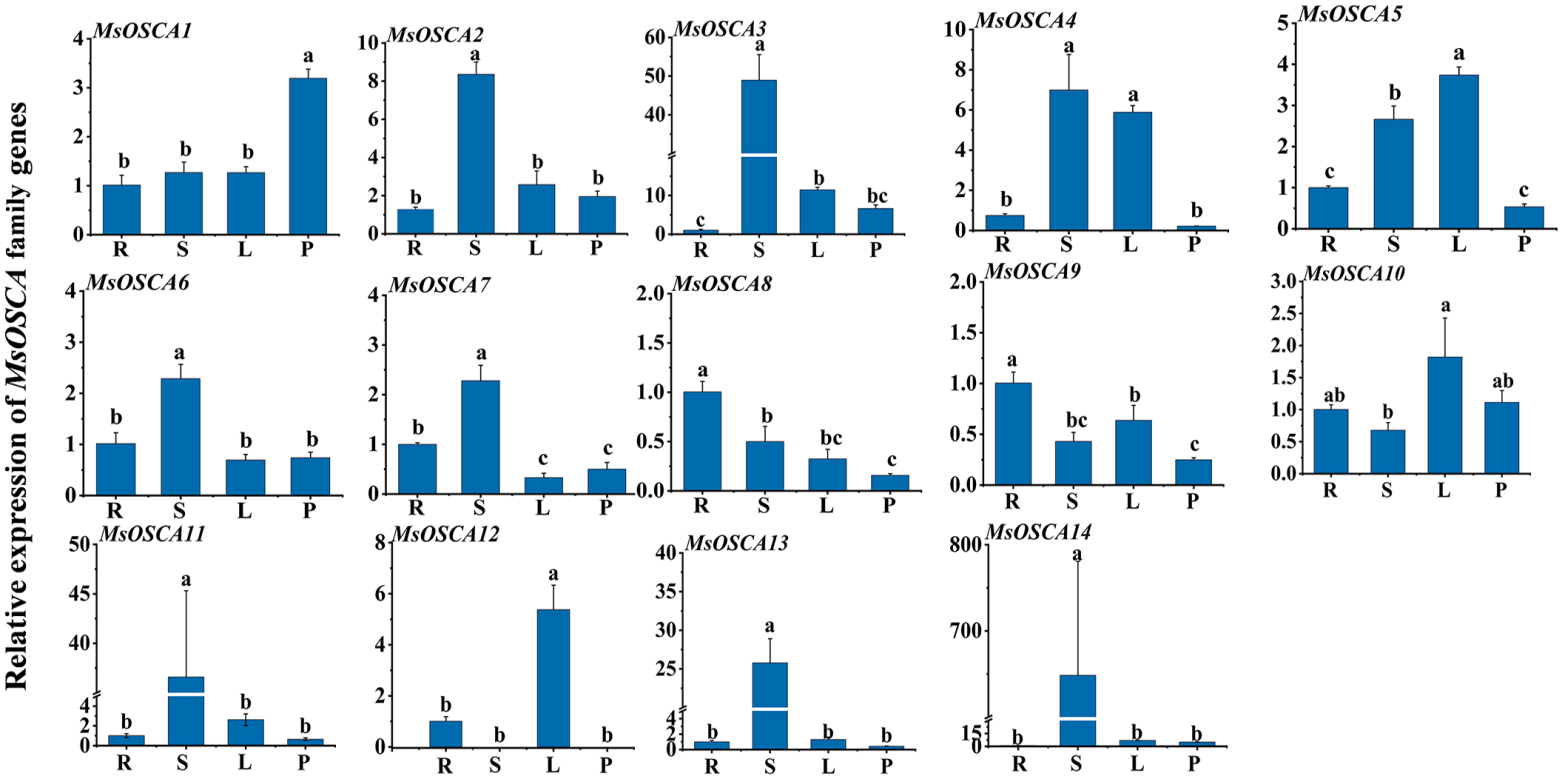
Relative expression levels analysis of *MsOSCA* genes in different tissues by RT-qPCR. Mean expression values relative to roots were calculated from three independent biological replicates and triple technique repetition. R, Root; S, Stem; L, Leaf; P, Petiole. Standard deviation is shown as bars above columns. Lower case letters indicate significant differences at *P* < 0.05.

We determined the relative expression levels of *MsOSCA* genes in roots, stems, leaves and petioles by RT–qPCR. As shown in **Figure 6**, the expression levels of *MsOSCA* genes varied considerably in four different tissues but were not completely consistent with the results of the transcriptome data. Except for *MsOSCA12*, other *MsOSCA* genes had different expression values in all tissues. The results showed that *MsOSCA12* had no detectable expression in stems and petioles. *MsOSCA2/-3/-6/-7/-11/-13/-14* had significantly higher expression levels in stems than in other tissues. The expression levels of the *MsOSCA5* and *MsOSCA12* genes were significantly higher in leaves than in other tissues. In addition, it is worth noting that *MsOSCA8* and *MsOSCA9* were highly expressed in roots (**Figure 6**). These results suggest that *MsOSCA* genes play different roles in the regulation of growth and development in alfalfa.

### Expression patterns of *MsOSCA* genes in response to abiotic stress

**Figure 7:**
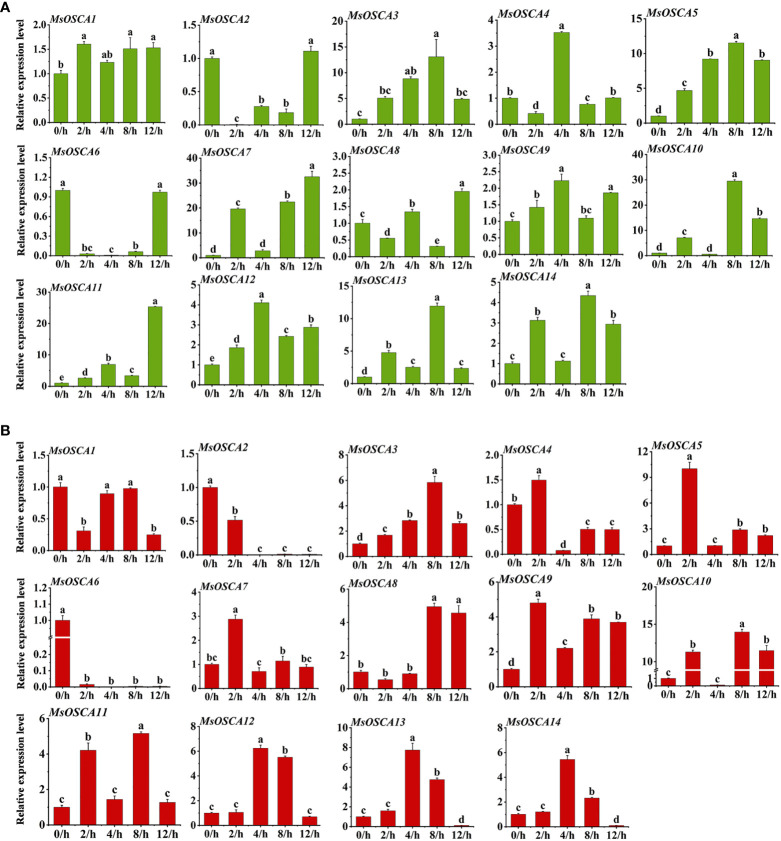
Relative expression levels analysis of *MsOSCA* genes under drought and salt treatments by RT-qPCR. **(A)** Relative expression levels of *MsOSCA* genes at 0, 2, 4, 8 and 12 h after drought treatment. **(B)** Relative expression levels of *MsOSCA* genes at 0, 2, 4, 8 and 12 h after salt treatment. Data represent the mean ± SD of three independent biological replicates and triple technique repetition. Standard deviation is shown as bars above columns. Lower case letters indicate significant differences at *P* < 0.05.

To investigate the potential function of *MsOSCA* genes under different abiotic stresses, the expression profiles of *MsOSCA* genes in alfalfa leaves under drought and salt stress were analyzed by RT–qPCR. With the exception of *MsOSCA2* and *MsOSCA6*, 12 *MsOSCA* members showed positive responses to drought stress under PEG-simulated drought stress conditions (**Figure 7A**). As shown in **Figure 7A**, the expression levels of the *MsOSCA3* and *MsOSCA5* genes initially increased and subsequently declined during drought stress. Further analysis showed that the *MsOSCA4* and *MsOSCA12* genes were dramatically upregulated at 4 h, with >3-fold of the control. The expression levels of *MsOSCA3/-5/-10/-13/-14* were more than 4-30-fold higher at 8 h than at 0 h. In the whole drought stress period, the expression levels of *MsOSCA7/-8/-11* genes showed the highest value at 12 h, and *MsOSCA7* was upregulated with an increase of >30-fold compared with the control. In addition, *MsOSCA2* and *MsOSCA6* decreased first and then increased under drought stress and finally maintained the same expression levels at 0 h (**Figure 7A**).

Compared with the control plants, all members of the *MsOSCA* gene family were differentially expressed under salt treatment, among which *MsOSCA1*, *MsOSCA2*, and *MsOSCA6* genes were significantly downregulated, and other genes were highly upregulated (**Figure 7B**). Among these differentially upregulated *MsOSCAs*, except for *MsOSCA8*, the expression of other genes showed an initial elevation and subsequent decrease under salt stress. The expression levels of *MsOSCA 12*, *MsOSCA13*, and *MsOSCA 14* reached their maximum at 4 h after salt stress, whereas the expression levels of *MsOSCA3*/-*8/*-*10*/-*11* reached their maximum at 8 h after salt stress (**Figure 7B**). In addition, the expression levels of the *MsOSCA5*, *MsOSCA7* and *MsOSCA9* genes were increased by 2-10-fold at 2 h under salt stress compared to 0 h. Compared with 0 h, the expression levels of *MsOSCA3*/-*8/*-*10*/-*11* were increased by 5-15-fold at 8 h. *MsOSCA10* and *MsOSCA11* showed similar expression trends under salt stress. However, *MsOSCA2* and *MsOSCA6* gene expression gradually decreased with the duration of salt stress (**Figure 7B**). Further analysis showed that *MsOSCA3*, *MsOSCA5*, *MsOSCA12* and *MsOSCA13* responded strongly to drought and salt stress. These results suggest that *MsOSCA* genes may play different roles under drought and salt stress.

### Expression patterns of MsOSCA genes in response to exogenous phytohormones

**Figure 8:**
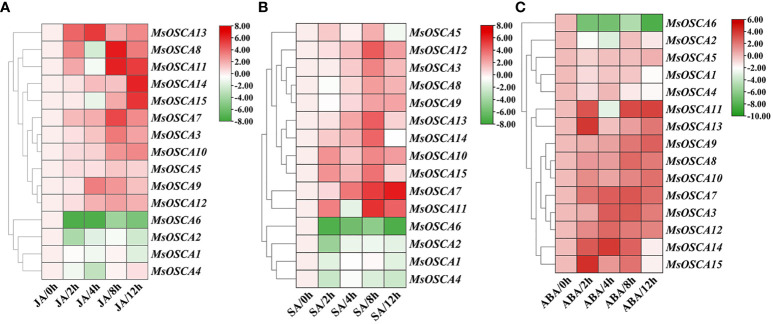
Expression patterns of *MsOSCA* genes under different phytohormone treatments. **(A)** Expression patterns of *MsOSCAs* under JA treatment. **(B)** Expression patterns of *MsOSCAs* under SA treatment. **(C)** Expression patterns of *MsOSCAs* under ABA treatment. The color scale at the right of the heatmap represents the log_2_ expression value, and the gradient from green to red represents low to high expression level.

Plant hormones are important stress signals because they stimulate the synthesis of phytohormones to regulate the expression of many stress-responsive genes in plants in response to abiotic stress (Putranto et al., 2015; Liu et al., 2022). To determine whether *MsOSCA* family genes were affected by different phytohormone treatments, the expression levels of *MsOSCA* genes were determined by RT–qPCR in the presence of JA, SA and ABA phytohormones (**Figure 8**; **Supplementary Table S5**). We found that the *MsOSCA* genes showed diverse expression patterns during JA treatment. Under JA stress, seven out of fourteen *MsOSCA* genes were significantly upregulated and peaked at 4, 8 or 12 h (**Figure 8A**). For example, the expression level of *MsOSCA12* at 4 h was 5-fold higher than that at 0 h. *MsOSCA3/-7/-8/-10* expression levels reached the highest at 8 h, while *MsOSCA13* and *MsOSCA14* expression levels were highest at 12 h, more than 5-fold higher than at 0 h. Interestingly, *MsOSCA8*, *MsOSCA10* and *MsOSCA14* showed initial fluctuation, with downregulated expression levels at 4 h. Furthermore, *MsOSCA1/-2/-4/-6* showed a decreasing trend expression pattern under JA treatment in alfalfa (**Figure 8A**).

The *MsOSCCA* expression levels were markedly triggered by SA treatment (**Figure 8B**). Most *MsOSCA* genes showed a positive response, and the expression levels of *MsOSCA3/-7/-8/-9/-11/-12/-14* increased significantly after treatment of alfalfa with SA (**Figure 8B**). In contrast, *MsOSCA1/-2/-4/-6* showed negative regulation in response to SA treatment. The expression levels of *MsOSCA8* first decreased slightly and then increased and peaked at 8 h. Further analysis showed that *MsOSCA3/-9/-11/-12/-14* expression reached a maximum at 8 h and was more than 3-fold higher than that of the control, while *MsOSCA7* expression was highest at 12 h and was 6-fold higher than that of the control (**Figure 8B**).

We found that the expression levels of *MsOSCA* genes were strongly associated with ABA hormone treatment, but the expression profiles varied in *MsOSCAs* (**Figure 8C**). The heatmap showed that the expression levels of nine genes (*MsOSCA-3/-7/-8/-9/-10/-11/-12/-13/-14*) were significantly upregulated in the presence of ABA, reaching the maximum expression values at 2 h, 4 h, 8 h and 12 h, respectively. In addition, *MsOSCA1*, *MsOSCA2* and *MsOSCA6* genes were downregulated, and *MsOSCA5* gene expression showed no significant change under ABA treatment (**Figure 8C**). Overall, the *MsOSCA7*, *MsOSCA10*, *MsOSCA12* and *MsOSCA13* genes were more likely to be strongly expressed under phytohormone treatments (**Figure 8**).

### MsOSCA protein interaction network prediction

To provide more evidence for the function of the MsOSCA proteins, we used the online STRING to predict the MsOSCA protein interaction network. The results showed that MsOSCA3 interacted with MsOSCA13 and MsOSCA14 (**Figure S4**). MsOSCA1 interacted with myelin transcription factor-like protein (A0JPZ2_ARATH) and transmembrane protein (Q5XV37_ARATH). MsOSCA3/-4/-5/-6/-13 interacted with ureide permease 4 (UPS4). In addition, MsOSCA13 interacted with PX domain-containing protein (EREL1), DnaJ homolog subfamily C (GRV2), and ADP-ribosylation factor (ARFC1).

### Subcellular localization of MsOSCAs

**Figure 9:**
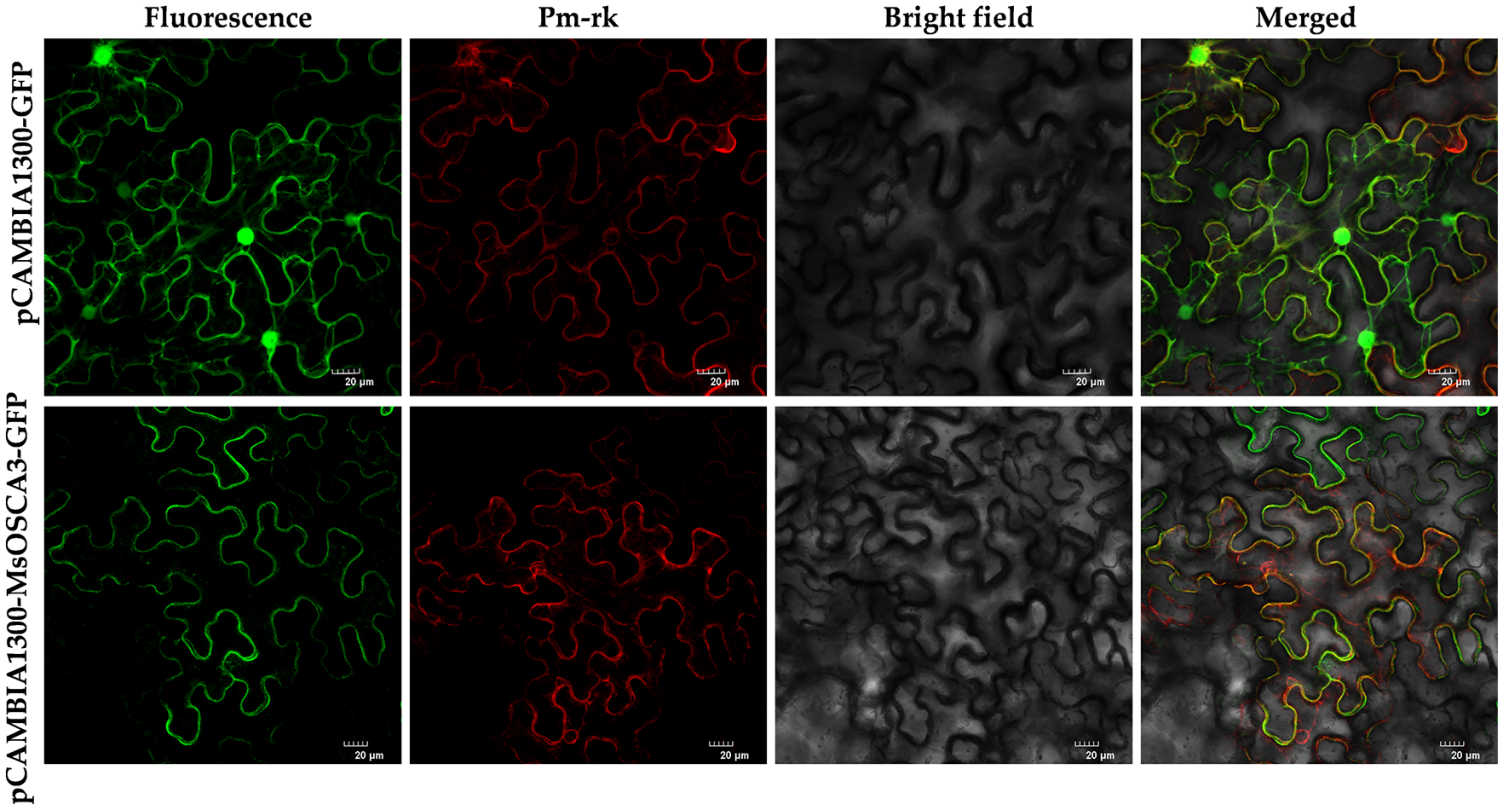
The subcellular localization of MsOSCA3 protein. pCAMBIA1300-MsOSCA3-GFP protein fusions transiently expressed in tobacco leaf cells. Excitation light wavelength: GFP field: 488 nm, PM-rk field: 587 nm. Scale bar, 20 μm.

Research on the subcellular localization of proteins is beneficial for deciphering their functions. As Ca^2+^ channels, OSCA family members are characterized by multiple transmembrane helices and are localized on membrane structures (Liu et al., 2022). To further verify the accuracy of subcellular localization prediction, the *MsOSCA3* gene, which was highly responsive to abiotic stress, was selected for transient expression assays in tobacco. In tobacco leaves injected with the control vector pCAMBIA1300-GFP, green fluorescence could be observed throughout the whole cell (**Figure 9**). However, in tobacco leaves injected with the pCAMBIA1300-MsOSCA3-GFP fusion vector, fluorescence was observed only in the plasma membrane. In addition, green fluorescence and red fluorescence could be merged by colocalization of the plasma membrane PM marker, indicating that MsOSCA3 encoded a membrane protein, which was in accordance with the predicted results.

## Discussion

OSCA proteins are regarded as osmotic sensors in plants and play an important role in sensing exogenous and endogenous osmotic changes and regulating plant growth, development and reproduction (Edel and Kudla, 2015; Xiaoyu et al., 2018). Genome-wide investigations and identification of *OSCA* gene families have been performed in most plant species. In this study, a total of 14 *OSCA* genes were identified in the alfalfa genome (**Table 1**), and this number was different from the number of *OSCA* genes in *Arabidopsis* (Yuan et al., 2014). The number of *OSCA* genes varied significantly between homologous species in systematic genome-wide analysis, which typically ranged from 11 to 15 in plants (Cai et al., 2022). However, 42 *OSCA* genes were identified in the wheat genome, far more than in any other species (Tong et al., 2021), indicating that the wheat *OSCA* gene family has undergone fragment and tandem duplication events during evolution.

The phylogenetic relationships between species are a fundamental part of biological research (Kapli et al., 2020). We systematically analyzed the phylogenetic relationships of OSCA proteins in alfalfa, *Arabidopsis*, *C. arietinum*, *V. radiata* and *C. cajan*. The OSCA proteins of alfalfa, *Arabidopsis*, *C. arietinum*, *V. radiata* and *C. cajan* were clustered into four phylogenetic clades (**Figure 1**), which was consistent with *Solanum habrochaites* (Miao et al., 2022), cucumber (Yang et al., 2022), *Gossypium hirsutum* (Yang et al., 2019), rice (Li et al., 2015) and maize (Cao et al., 2020), possibly due to the conservative evolution of *OSCA* genes. In addition, on each clade had AtOSCAs, MsOSCAs, CcOSCAs, CaOSCAs and VrOSCAs, suggesting that they might have a common ancestor. The OSCAs of alfalfa and *C. arietinum* were close to each other in clades I and IV, indicating that these genes were highly homologous. The functional domains of genes are important factors in the regulation of element activity and gene expression. The RSN1_7TM functional domain is the signature domain of the OSCA family. Our results showed that all MsOSCA proteins contained the RSN1_7TM functional domain (**Figure 2**), which was consistent with maize (Cao et al., 2020), barley (Cai et al., 2022) and legumes (Chakraborty et al., 2023). In addition, some proteins (MsOSCA1/-2/-3/-4/-6/-7/-9/-10/-13) of the MsOSCA family contained RSN1_TM and PHM7_cyt functional domains, similar to wheat (TaOSCA-1/-2/-3/-4/-14/-27) (Tong et al., 2021). This may explain the functional variability of the *OSCA* genes. The structure of exons and introns affects the function of proteins and the expression and regulation of genes, which means that exons and introns enable genes to play an important role in organisms and thus in the maintenance of life (Xu et al., 2012). *MsOSCA* genes had different numbers of exons/introns, with some genes having similar exon/intron structures but different sequence lengths, which was similar to those of other plants, such as maize (Cao et al., 2020), *Triticum aestivum* (Tong et al., 2021) and *Populus tomentosa* (Jie et al., 2022). *Cis*-acting elements within the gene promoter region play a crucial role in the regulation of gene expression. The different types of *cis*-acting elements in the gene promoter predict that the gene may have different functions in response to stress (Chen et al., 2015a). Analysis of *cis*-acting promoter elements revealed that *MsOSCAs* contained elements involved in hormone responses (JA, SA, ABA and GA), photoresponses, metabolic regulation and stress responses (low temperature, drought) (**Figure 3**), suggesting that *MsOSCA* genes may be involved in the regulation of abiotic and biotic stress responses and plant hormone transduction. For example, the *MsOSCA3*, *MsOSCA5* and *MsOSCA11* genes contained MBS elements, suggesting that they may be active in drought stress. The *MsOSCA1*, *MsOSCA10* and *MsOSCA12* genes had multiple hormone response elements, indicating that they may play a role in the hormone response and regulation of various physiological metabolic processes in plants. Previous studies have indicated that some *OSCA* genes are involved in hormonal or abiotic stress responses when specific *cis*-elements are found in their promoter regions, such as *HvOSCA4.1* (She et al., 2022) and *ZmOSCA2.5* (Cao et al., 2020). This was in agreement with our predictions. Protein interaction predictions showed that MsOSCA3 interacted with MsOSCA13 and MsOSCA14, suggesting that the three proteins may be functionally similar (**Figure S4**). MsOSCA1 interacted with myelin transcription factor-like protein (A0JPZ2_ARATH) and transmembrane protein (Q5XV37_ARATH), and we speculated that they function through interactions. These data provide a basis for further studies of the function of MsOSCA proteins.

In this study, the *MsOSCA* genes were distributed on each chromosome of alfalfa, but their distribution was not uniform, which is similar to that in most species (Jie et al., 2022; Yang et al., 2022). Gene duplication is one of the most important driving forces for the evolution of the genome and the genetics of a species (Magadum et al., 2013). The two main causes of gene family amplification in plants are segmentation and tandem duplication (Lawton-Rauh, 2020). Gene duplication can not only add new members to the gene family but also enrich the function of the gene family, which greatly promotes the genetic evolution of various organisms (Du et al., 2019). Tandem and segmental replication events were observed in the *MsOSCA* gene family (**Figure 4A**), indicating that gene replication was also the driving force for the expansion of *MsOSCA* gene family members. Previous studies have shown that gene pairs with high homology in the gene family may have collinearity, which makes them have similar biological characteristics and expression patterns (Jia et al., 2021). In the present study, we found that *MsOSCAs* had collinearity with *Medicago truncatula* and *Arabidopsis*, suggesting that they might have similar functions, which requires further investigation. However, there were more collinear gene pairs between alfalfa and *Medicago truncatula* than between *Arabidopsis*, indicating higher homology between them, which provides a basis for predicting the expression and function of *MsOSCA* gene*s*.

The tissue specificity of genes is of great importance for the study of life processes and protein functions in tissues (Hwang et al., 2011). Although the tissue specificity of the *OSCA* gene family has been identified in many plants, the expression level of *OSCA* genes has significantly changed in different tissues with the evolution of species (She et al., 2022). In this study, we found that all *MsOSCA* genes of alfalfa had tissue-specific expression (**Figure 6**), similar to the *AtOSCA* and *CsOSCA* gene families (Yuan et al., 2014; Yang et al., 2022). *MsOSCAs* were expressed in both roots and leaves, indicating that they might play important roles in vegetative growth. However, the expression of *MsOSCA12* was not detected in the stems and petioles (**Figure 6**), and transcriptome data analysis showed that *MsOSCA12* was only expressed in flowers (**Figure 5**), so we hypothesized that the gene may be related to circadian rhythm, osmotic stress sensing site and reproductive growth and development (Cai et al., 2022; Miao et al., 2022). *MsOSCA8* was significantly more highly expressed in roots than in other tissues, which may be closely related to the perception and response of osmotic stress. The tissue expression of *MsOSCA* genes was not completely consistent with the transcriptome results, which may be caused by different alfalfa varieties. These results suggest that the expression of the *OSCA* gene family in alfalfa is tissue specific.

Drought and salt stress are the most common abiotic stresses encountered by plants. Plants have evolved to adapt to biotic or abiotic stresses by inducing the expression of stress-related genes to modulate physiological response processes and thus improve stress tolerance (Gong et al., 2020; Kaur et al., 2022). In recent years, it has been shown that *OSCA* genes play important roles in the regulation of plant responses to osmotic stress (Xiaoyu et al., 2018; Tong et al., 2021). In this study, in addition to *MsOSCA2* and *MsOSCA6*, 12 *MsOSCA* members showed positive responses to drought stress (**Figure 7A**), indicating that *MsOSCA* genes may have a regulatory function in osmotic stress. In addition, except for *MsOSCA1*, *MsOSCA2* and *MsOSCA6*, the expression levels of other genes were significantly increased under salt stress (**Figure 7B**), suggesting that these genes responded positively to salt stress. Our in-depth analysis revealed that *MsOSCA3, MsOSCA5, MsOSCA12* and *MsOSCA13* were remarkably upregulated under both drought and stress (**Figure 7**), which was consistent with the predicted results of the promoter regulatory elements. *MsOSCA10* and *MsOSCA11* had similar expression trends under salt stress, which correlated with their close relationship in the phylogenetic tree. Researchers found that the *ZmOSCA2.4* gene was significantly upregulated in response to drought and salt stress, and overexpression of *ZmOSCA2.4* significantly enhanced drought tolerance in *Arabidopsis* (Cao et al., 2020). The positive regulatory role of the *GhOSCA1* gene under salt and drought stress was demonstrated by the increased sensitivity of *GhOSCA1.1*-silenced plants to salt and drought stress (Yang et al., 2019). Therefore, we speculate that *MsOSCA* genes may be involved in the regulation of drought and salt-induced osmotic stress.

Phytohormones play important roles in plant growth, development and stress responses (Chen et al., 2015b; Zhao et al., 2021). Appropriate accumulation of multiple hormones in plants can improve the tolerance level of plants to abiotic and biotic stresses (Singh and Roychoudhury, 2023). JA acts as a signaling molecule that regulates plant responses to biotic and abiotic stresses and induces resistance gene expression (Ruan et al., 2019). SA can counteract the negative effects of abiotic stress on plants (Colebrook et al., 2014). ABA improves plant tolerance to adverse conditions by regulating stomatal opening and closing and the expression of stress response genes (Chen et al., 2014). The *OSCA* gene family has been reported to be involved in regulating multiple hormone responses (Putranto et al., 2015; Xiaoyu et al., 2018). For instance, the *HvOSCA2.2*, *TaOSCA12* and *TaOSCA39* genes exhibited strong responses under ABA stress (Tong et al., 2021; Chakraborty et al., 2023). In this study, all *MsOSCAs* showed positive responses to JA and SA stress except *MsOSCA1/-2/-4/-6* (**Figures 8A, B**). Most *MsOSCA* gene*s* were upregulated under ABA treatment, while *MsOSCA1*, *MsOSCA2* and *MsOSCA6* were downregulated. Moreover, the *MsOSCA7*, *MsOSCA10*, *MsOSCA12* and *MsOSCA13* genes were more likely to be highly expressed under ABA treatment (**Figure 8C**). These results indicated that the expression of most *MsOSCA* genes was consistent with the results of promoter element analysis, implying that these genes may play an important role in the hormone signaling pathway. Similarly, the expression levels of *BnOSCA1.1a*, *BnOSCA1.1b* and *BnOSCA3.1c* were relatively higher under ABA treatment, indicating that these three *BnOSCAs* respond positively to ABA hormone treatment (Zhang et al., 2023). The subcellular localization of MsOSCA3 on the plasma membrane was consistent with that of some OSCA proteins, such as GmOSCA2.3, GmOSCA3.2 (Liu et al., 2022) and OsOSCA4.1 (Zhai et al., 2020), which further confirmed that the MsOSCA protein may play a vital role in the cell membrane.

## Conclusion

In this study, we performed a genome-wide scale analysis of *OSCA* genes in alfalfa. A total of 14 *OSCA* genes were identified in the alfalfa reference genome, and the basic molecular characteristics, evolutionary relationships, motifs, gene structure, and duplication events were investigated. The *MsOSCA* genes were classified into three clades, all of which shared the DUF221 functional domain. Alfalfa was closely related to *Arabidopsis* and *Medicago truncatula*, and gene duplication events were found in the *MsOSCA* gene family. The promoter regions of *MsOSCAs* contained a large number of abiotic stress and hormone response elements, such as MBS, ABRE, P-box and TCA elements. The expression of *MsOSCA* genes in alfalfa had tissue specificity. *MsOSCA* genes could strongly respond to abiotic stress, indicating that *MsOSCA* genes play important roles in regulating the response of plants to various abiotic stresses. These results will provide the basis for further studies on the function of *MsOSCA* genes under abiotic stress.

## Data availability statement

The datasets presented in this study can be found in online repositories. The names of the repository/repositories and accession number(s) can be found in the article/**Supplementary Material**.

## Author contributions

XL: Conceptualization, Investigation, Methodology, Writing – original draft. XW: Investigation, Methodology, Writing – review and editing. XM: Investigation, Writing – review and editing. WC: Investigation, Writing – review and editing. YL: Investigation, Writing – review and editing. WS: Investigation, Writing – review and editing. BF: Conceptualization, Funding acquisition, Writing – review and editing. SL: Conceptualization, Funding acquisition, Methodology, Supervision, Writing – review and editing.
