# Supplementary material for: Genome-wide investigation and expression analysis of OSCA gene family in response to abiotic stress in alfalfa
Source: Front Plant Sci. 2023 Nov 3;14:1285488. doi: 10.3389/fpls.2023.1285488 (PMC10655083; doi:10.3389/fpls.2023.1285488)
Supplement: Supplementary file 1 [file DataSheet_1.pdf]

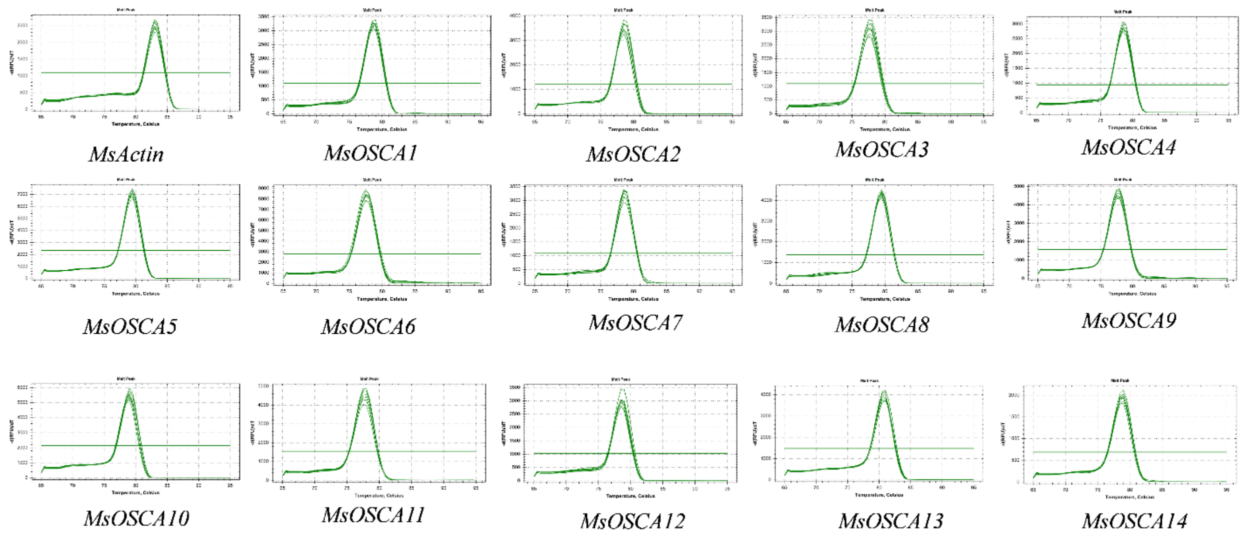

Figure S1 Melting profiles of all primers

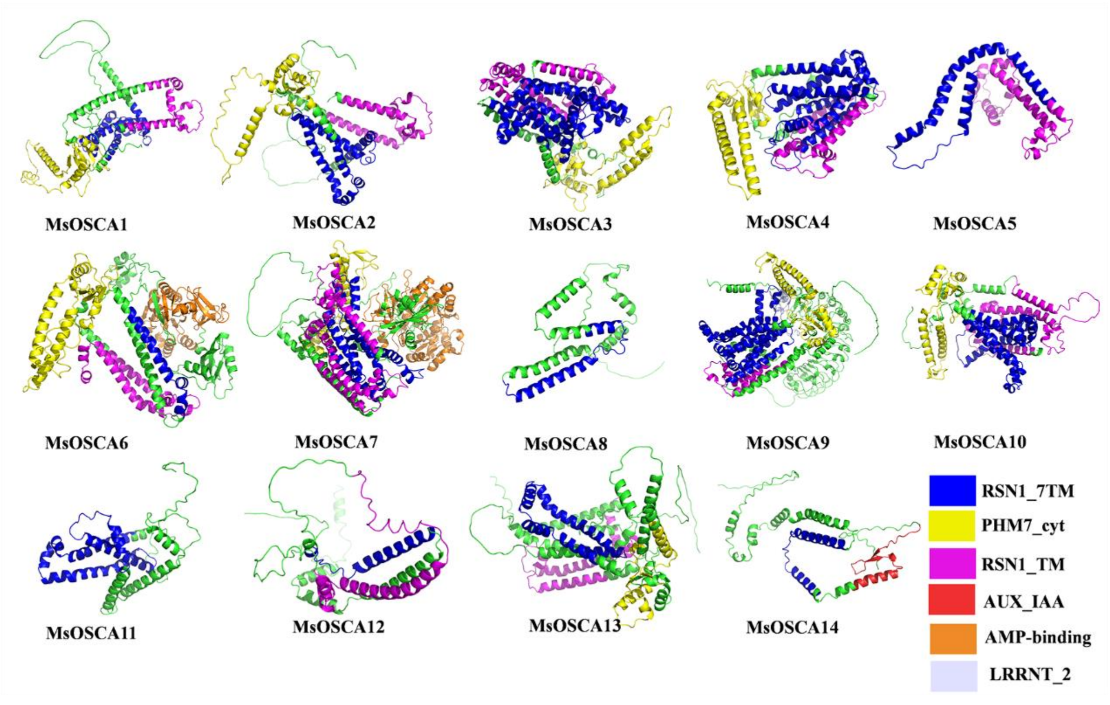

Figure S2 3D structure and functional domains of MsOSCA proteins

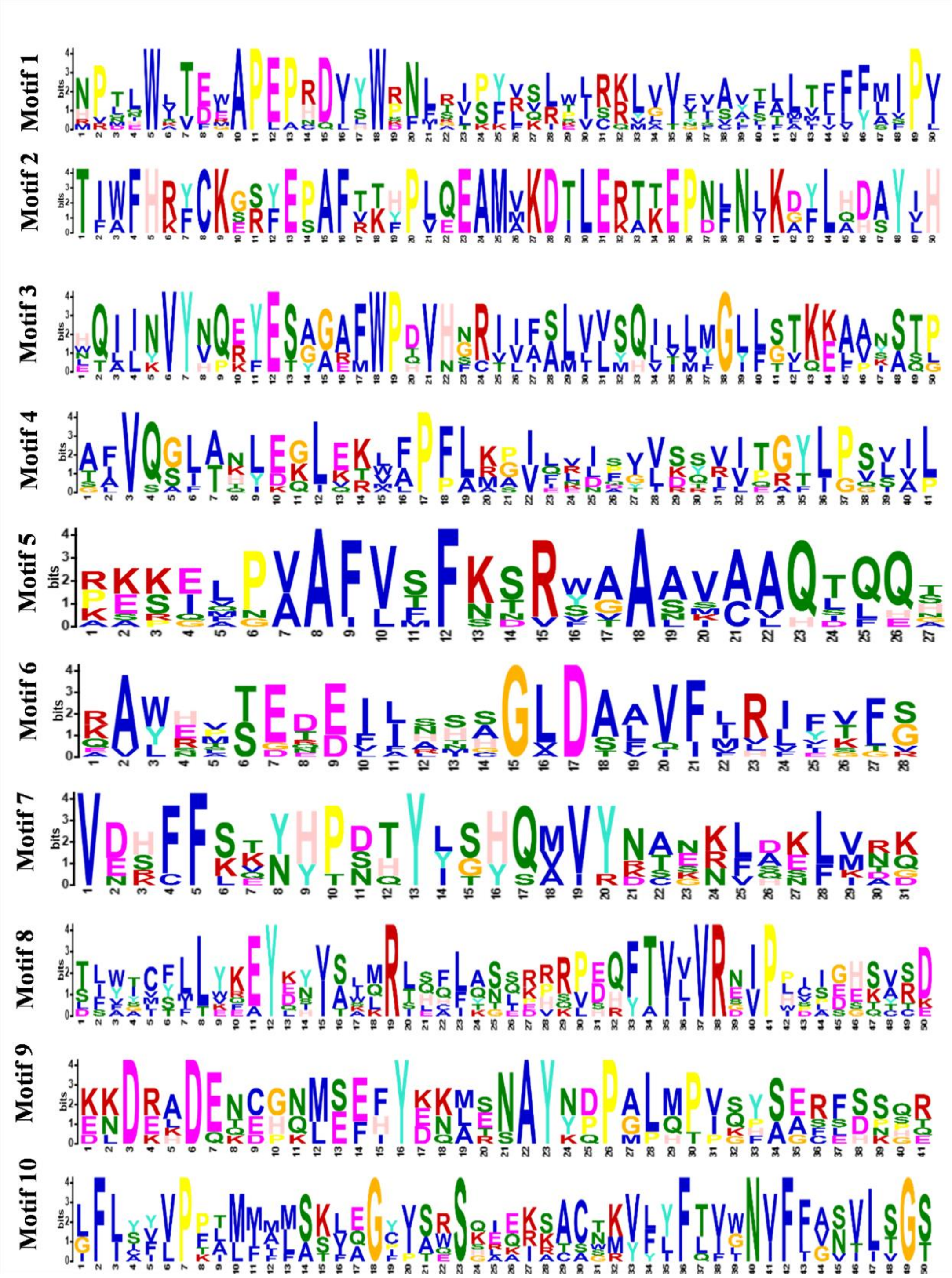

Figure S3 The MEME motifs of MsOSCA proteins

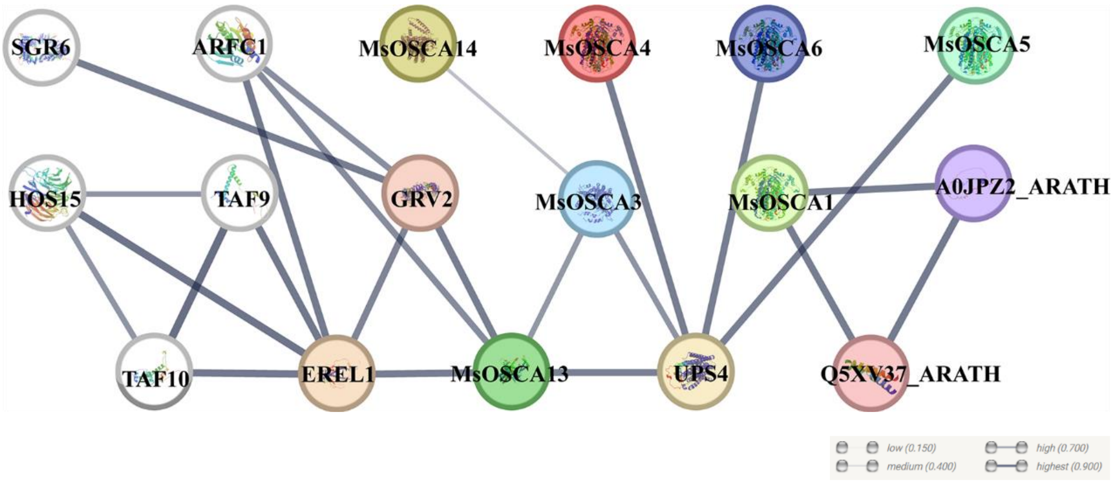

Figure S4 MsOSCA Proteins Interaction Network
